# Inferring long-distance connectivity shaped by air-mass movement for improved experimental design in aerobiology

**DOI:** 10.1038/s41598-021-90733-2

**Published:** 2021-05-27

**Authors:** Maria Choufany, Davide Martinetti, Samuel Soubeyrand, Cindy E. Morris

**Affiliations:** 1 INRAE, BioSP, 84914 Avignon, France; 2 INRAE, Pathologie Végétale, 84914 Avignon, France

**Keywords:** Microbiology, Plant sciences, Mathematics and computing, Computational biology and bioinformatics, Network topology, Statistical methods, Atmospheric dynamics

## Abstract

The collection and analysis of air samples for the study of microbial airborne communities or the detection of airborne pathogens is one of the few insights that we can grasp of a continuously moving flux of microorganisms from their sources to their sinks through the atmosphere. For large-scale studies, a comprehensive sampling of the atmosphere is beyond the scopes of any reasonable experimental setting, making the choice of the sampling locations and dates a key factor for the representativeness of the collected data. In this work we present a new method for revealing the main patterns of air-mass connectivity over a large geographical area using the formalism of spatio-temporal networks, that are particularly suitable for representing complex patterns of connection. We use the coastline of the Mediterranean basin as an example. We reveal a temporal pattern of connectivity over the study area with regions that act as strong sources or strong receptors according to the season of the year. The comparison of the two seasonal networks has also allowed us to propose a new methodology for comparing spatial weighted networks that is inspired from the small-world property of non-spatial networks.

## Introduction

Organic particles are ubiquitous in the air^1–3^ and may originate from very different sources^4^, such as plant canopies^5^, soils^6^, urban areas^7, 8^ or surface waters^9^. Despite their relative sparse density with respect to the volume of an air mass, their presence and transportation across the planet has proven to have strong effects on many phenomena such as colonization and invasions by plants and insects^10–14^, human, animal or plant epidemics^15–23^ and atmospheric processes^24–26^. Some of these particles can be transported through the atmosphere over hundreds or even thousands of kilometers^1, 27, 28^, depending on their shape and mass^29^. Furthermore, seasonal trends of microbial compositions in the air have been repeatedly observed in several studies, especially in temperate climates^30–38^. All these factors make it particularly difficult to disentangle the complexity of the biological composition of air samples, that may include hundreds of species from both local and distant sources, and that can vary drastically over the course of a year.

During the last decades, an increasing number of studies has addressed this problem by collecting and analyzing air samples with a plethora of experimental settings that varied enormously from one study to the other in the number and choice of sampling locations, duration and frequency of sampling, type of air sampler, among others^39^. Regardless of these differences, most of these large-scale studies have in common a certain lack of forethought in the choice of sampling locations and dates, that are often dictated by logistical convenience, instead of relying on previous knowledge of air-mass sources (whereas prevailing winds, for instance, are classical input for the design of sampling in small-scale dispersal surveys^40, 41^). Nonetheless, there are a few exceptions in which the authors reconstructed the geographical origins of the air mass by looking at backward trajectories associated with the air masses from which they have collected their samples^30, 31, 35, 42^.

Here we present a framework to identify stable and recurrent connections between distant portions of a territory via air-mass movements on large spatiotemporal scales. A priori knowledge of the location and seasons of occurrence of such aerial connections would provide very useful rational for designing the layout of air sampling schemes for early detection of airborne propagules of invasive plant and insect species, microbial pathogens and their vectors. We considered the air-mass connectivity across the coast of the Mediterranean basin. The similar climates and vegetation along the northern and southern coasts of the Mediterranean basin would increase the likelihood of survival of propagules that successfully migrated across the sea. The rather large body of water between the coasts will facilitate demonstrating events of long-distance aerial dispersal by limiting the number of potential intermediate sources. Importantly, the Mediterranean basin is also a hot spot for spread of human and plant diseases where efforts to survey for invasions and emergences are intensifying. For example, there is concerted effort to survey for insect vectors of zoonotic diseases^43^. This surveillance, which focuses mostly on traditional ground-based observations, can be strengthened in light of recent findings that some insects such as mosquitos are indeed transported hundreds of kilometers by air mass movements at tens to hundreds of meters above ground^14^. This raises the question of the most opportune sites and times for observing this long-distance dissemination.

To identify recurrent patterns that could optimize air sampling effort, we first partitioned the study area into uniform regions and estimated their pairwise degree of connectivity, regardless of their geographical distance, based on the presence of recurrent air-mass trajectories between them. We naturally resorted to network theory, since it allows to represent complex connectivity patterns with the formalism of nodes and links and can further be exploited to infer new and interesting properties of the graphs^44^. We identified two seasonal patterns, one in summer and the other in winter, with relatively distinct behaviours. To characterize the two seasonal networks, we also proposed a new method of comparing spatial weighted and directed networks that accounts simultaneously for the geographical distance and the edge weight between nodes. Finally, we used different indices to assess the relevance of nodes within the network and their likelihood of infecting and/or being infected during a simulated susceptible-infected (SI) epidemic on the network. This allowed us to identify sets of influential spreaders and strong receptors, which can be used for the design of efficient epidemic surveillance strategies.

## Results

### Description of the case study and data collection

Daily 48-h backward air-mass trajectories from January 1, 2011 to December 31, 2017 were extracted using the HYSPLIT software^45^ from a set of 604 arrival points chosen across the study area. The arrival points of the air-mass trajectories, that represent the location of the nodes of the constructed spatial networks, correspond to the centroids of a grid with mesh size of 74 km covering the coastline of the Mediterranean Sea from 5 km up to 250 km inland from the coast and including the four largest islands (namely Sicily, Sardinia, Cyprus and Corsica) and the Balearic archipelago^44^. The total number of computed trajectories was 1,543,220.

### Network construction and properties

We constructed spatiotemporal networks with discrete (daily) timestamps whose nodes represent the cells of the mesh described earlier. An edge between two nodes *i* and *j* at timestamp *t* is equal to one if the air-mass trajectory arriving at cell *j* at day *t* has passed over the cell *i* during the previous 48 h. This simple method allowed us to construct 2555 ($365 \times 7$) spatial directed networks, but we needed a way to downscale this complex information to fewer, longer periods of time. The first approach was to concentrate the 2555 daily networks into a single projected static network by averaging the number of connections between each pair of nodes (referred hereafter as 2011–2017 network). Furthermore, we projected the 2555 daily networks at yearly and monthly cadence, i.e. averaging all the networks of the year 2011, then year 2012, etc. and averaging all the networks of the 7 months of January, then all the networks of the 7 months of February, etc. We then computed different network metrics (diameter, density, transitivity, average shortest path and degree correlation) as reported in Table 1 of the Appendix. The comparison of the metrics for the 7 yearly networks showed no particular trend, meaning that the average yearly behaviour has not changed during the 2011–2017 period. On the other hand, the comparison between the metrics of the 12 monthly-averaged networks highlighted a clear seasonal pattern. We hence used a hierarchical clustering method based on the Cut distance between the 12 monthly networks (see^46^ and “Methods” section for the details) in order to confirm the observed patterns. The clustering algorithm identified two main seasons: summer (from May to September) and winter (from October to April) that have hence been gathered into two projected static networks whose network metrics are shown in Table 1 (referred hereafter simply as summer and winter networks). We can observe that the summer network has a significantly larger diameter (intended as the length of the longest of all the calculated shortest path) than the winter network, while it has a lower density (ratio between the sum of all edge weights and the number of all possible edges). Also, the summer network has the longest average shortest path between any couple of nodes and lower transitivity (e.g. the average probability that the adjacent nodes of a node are also connected).

**Table 1:** Network metrics for the three networks representing the average connectivity during the entire period 2011–2017, and the summer and winter seasons.

	Diameter	Density	Transitivity	Degree correlation	Average shortest path
2011–2017	37.5	0.28	0.74	0.306	2.06
Winter	45.4	0.24	0.73	0.171	2.32
Summer	58.4	0.19	0.68	0.281	2.57

**Figure 1:**
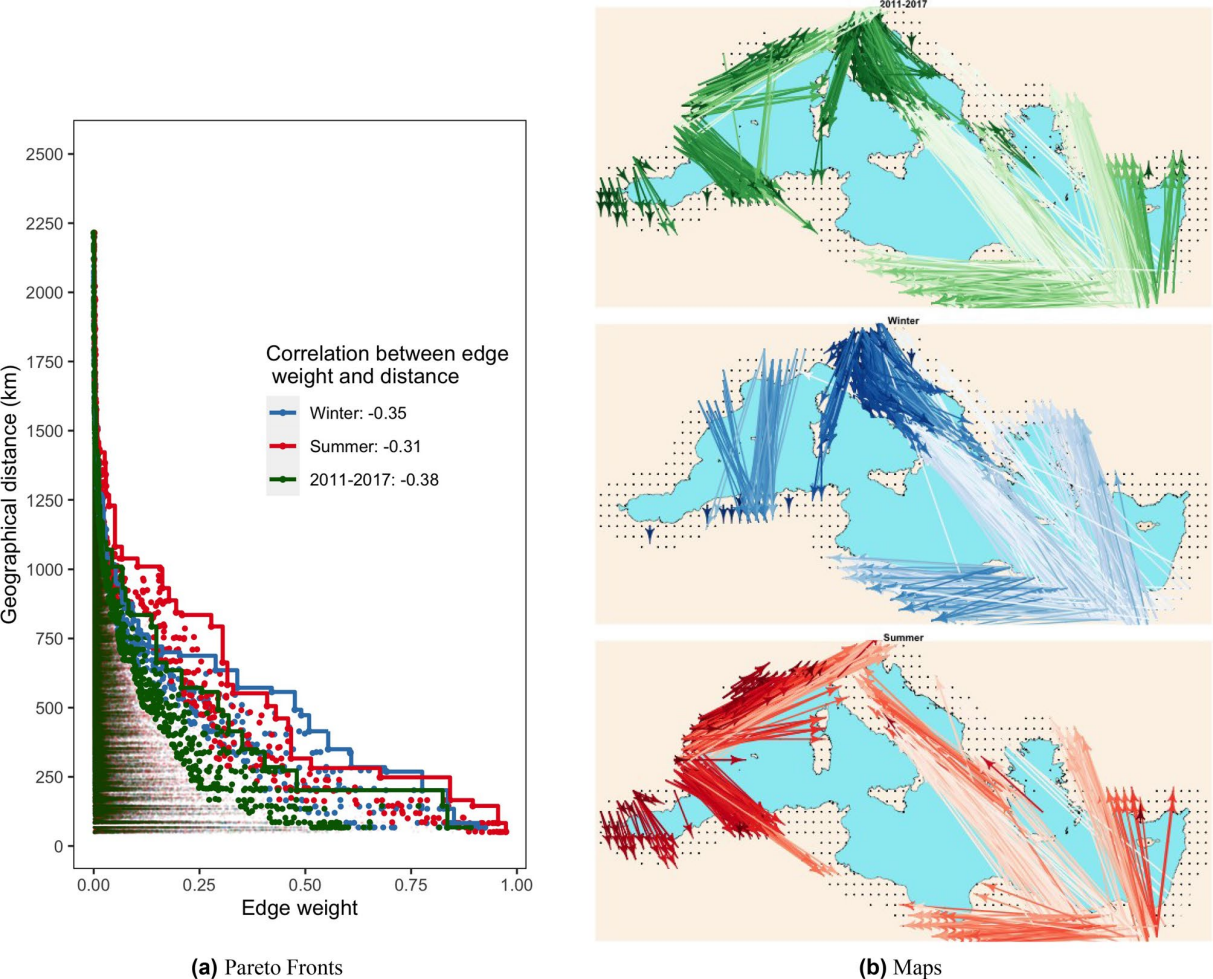
(**a**) The distribution of weights and distances for all pair of non-null edges of the 2011–2017, summer and winter networks. The Pareto fronts and the 1% of the edges that lie closest to the Pareto fronts are depicted with a bigger dot. (**b**) The 1% of the edges that lie closest to the Pareto fronts, where the intensity of the color correspond the strength of the weight.

One drawback of the metrics used so far is that they only account for the topological properties of the weighted networks, while they overlook the spatial signature that is inherently associated to the graph. The most natural consequence of considering the spatial structure of the network is that we expect geographically close nodes to be more strongly connected than nodes that are farther apart, a phenomenon that is also known as the Tobler’s first law of geography^47^. Indeed, we found that for the three considered networks, the correlation coefficients between edge weights and distances are always negative (0*.*38 for 2011–2017, 0*.*35 for winter and 0*.*31 for summer), meaning that stronger weights are associated with shorter distances and, vice versa, weaker weights are associated to longer distances. Furthermore, consider the case of three nodes (*i, j, k*) such that the weight of the edge between *i* and the other two nodes is the same (*E*(*i, j*) = *E*(*i, k*)), while their geographical distance is significantly different, e.g. $d\left(i,j\right)\gg d(i,k)$. The fact that the weight between *i* and *j* is the same as between *i* and *k*, even if *i* and *j* are much further apart in space than *i* and *k* may be of great relevance in certain applications. These two considerations imply that in a spatial weighted network, certain edges are more prominent than others, in particular those that maximize, at the same time, weight and geographical distance. Here we introduce a new way of analysing networks that accounts for these aspects. It allows, first, to compare multiple spatial weighted networks, and second, to improve the visualization by pruning the number of edges hence avoiding the so-called ’hairball’ effect^48^ (overly dense representation of edges that makes the network undecipherable). In the left panel of Fig. 1 we depicted the weights and distances of all non-null edges of the 2011–2017, summer and winter networks, also highlighting the Pareto fronts that maximize both quantities. We can observe that summer network is capable of longer and stronger connections than the winter network (particularly for nodes that are more than 700 km apart), while the winter network has generally stronger connections in the range between 300 and 500 km. In Fig. 1b we mapped the directed edges that correspond to the 1% of points that lie closest to the Pareto front of Fig. 1a. We can observe that the edges of the winter network with higher weights and small to medium distances tend to align with the Italian peninsula from North to South plus some edges from France and Spain towards Algeria, while longer edges cross the Mediterranean from South-East to North. Interestingly, we notice that nodes in Southern Italy receive edges both from Northern Italy (medium distance) and from Northeastern Africa (very long distance). For the summer network we observe that most of the short and medium distance edges start from the Western European coast (France and Spain) and travel eastward towards Northwestern Africa (short distance and strong weight) and Northern Italy, Corsica and Slovenia (medium distance and weight), while all long-distance edges connect Greece, Lybia and Egypt with Italy and Turkey. As expected, the 2011–2017 network integrates both summer and winter networks characteristics, with a seasonal stability that can be observed in the Eastern Mediterranean with recurrent connections from South-East to North, West and North-West.

### Indices of the relevance of nodes on network structure

**Figure 2:**
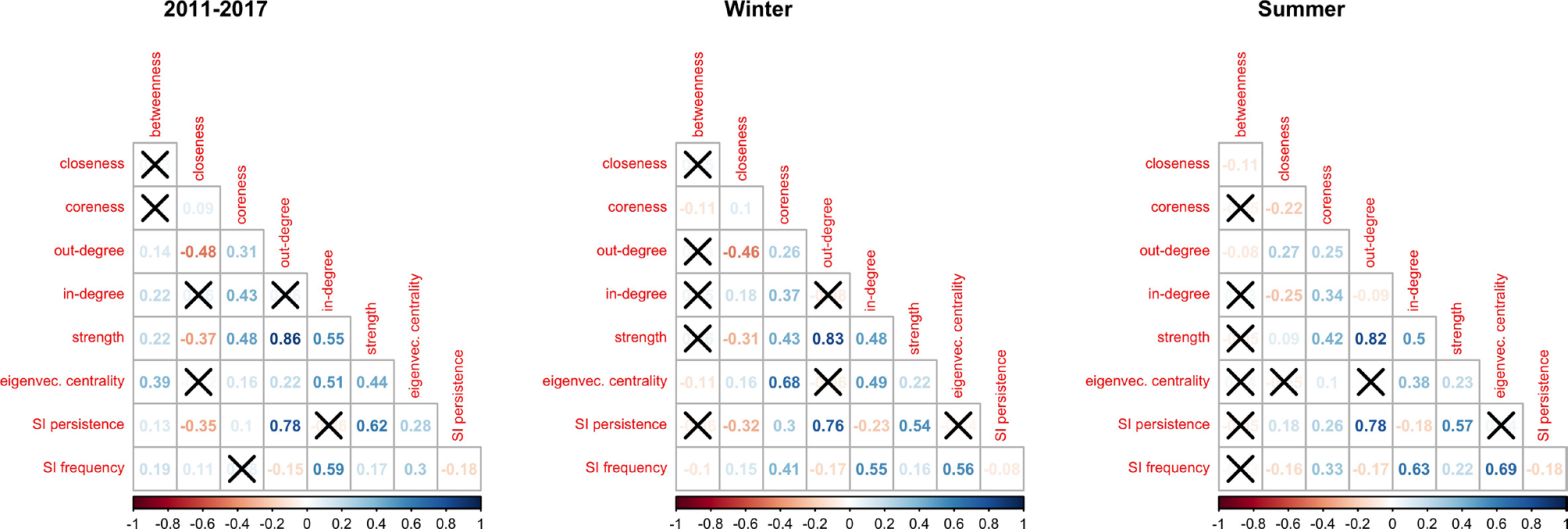
Correlation plots of node indices. All reported correlation coefficients are statistically significant at 95% confidence level.

We computed local node properties of the 2011–2017, summer and winter networks. Local node properties measure the relevance of single nodes within a network with respect to certain dynamics, such as the flow of information or the spread of a disease and their centrality with respect to the topology of the rest of the network. We hence computed different node relevance indices in order to identify those nodes that play a prominent role in the network, namely betweenness centrality, closeness centrality, coreness centrality, eigenvector centrality, out-degree, in-degree, strength (see “Methods” section for the details). Furthermore, we simulated an SI epidemic spread starting from each node and recorded two more indices, namely SI persistence and SI frequency, that indicate, for every node *i*, the percentage of nodes that will be infected when an epidemic starts from *i*, and the percentage of times that node *i* has been infected across all simulated epidemics (see “Methods” for the details on the simulated SI epidemic model). Since the SI persistence index measures the likelihood of a node to spread the disease in case it is the outbreak node, it is expected to be related to other indices representing its outreach capacity and, indeed, we can observe in Fig. 2 that the SI persistence index is positively correlated with the out-degree (0*.*78, 0*.*76 and 0*.*78 for the 2011–2017, winter and summer networks, respectively), the strength index (0*.*62, 0*.*54 and 0*.*57) and the coreness centrality index (0*.*10, 0*.*30 and 0*.*26), while the correlation with the other indices is less straightforward. On the other hand, the SI frequency index measures the likelihood of a node of been infected by an epidemic that started somewhere else in the network, hence we expect it to be correlated with centrality measures, as we can observe in Fig. 2, where we found that it is always positively correlated with the in-degree (0*.*59, 0*.*55 and 0*.*63 for the 2011–2017, winter and summer networks, respectively), the strength index (0*.*17, 0*.*16 and 0*.*22) and the eigenvector centrality index (0*.*30, 0*.*56 and 0*.*69) and negatively correlated with the out-degree (− 0*.*15, − 0*.*17 and − 0*.*17).

**Figure 3:**
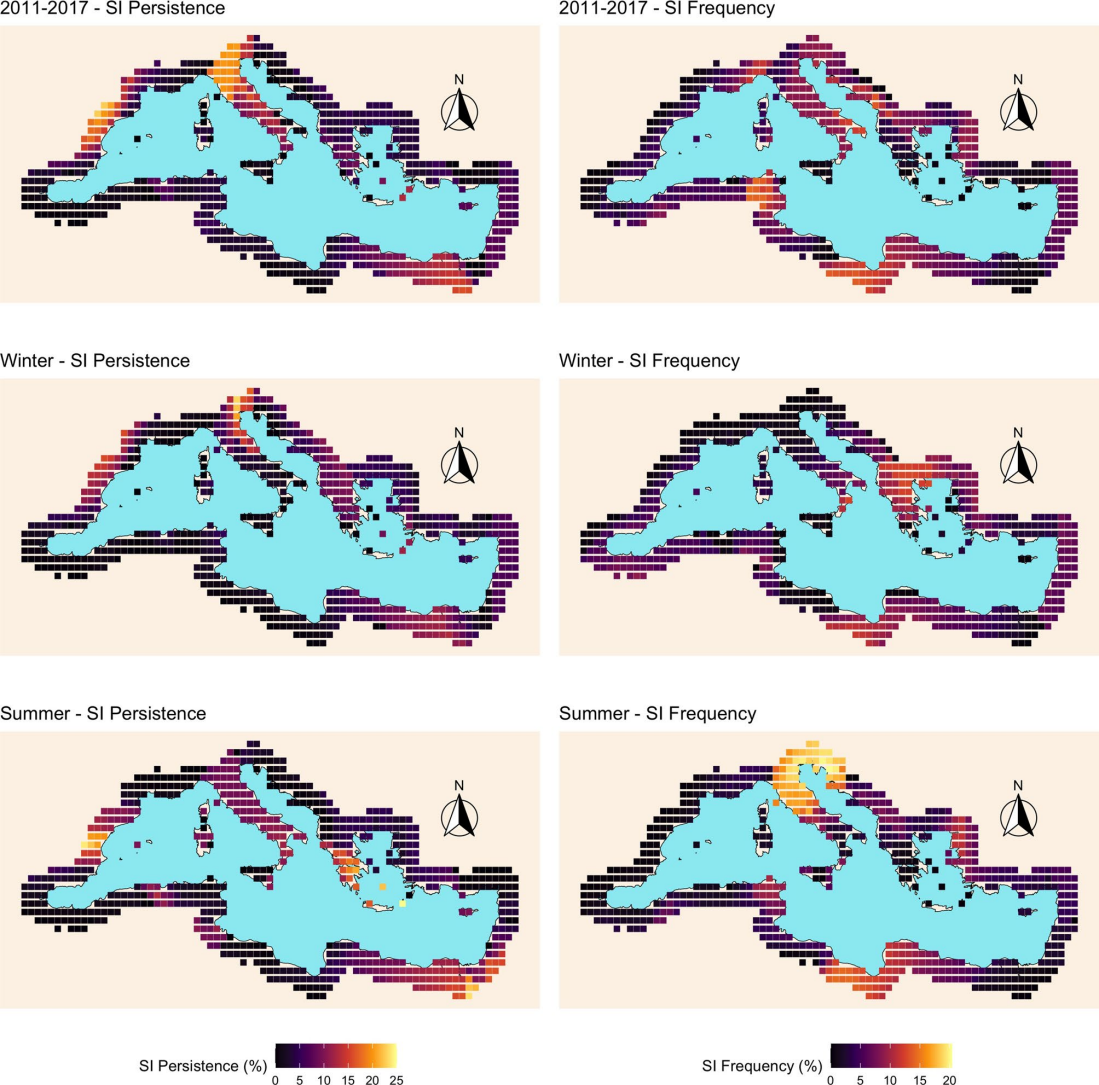
SI persistence and SI frequency.

Finally, Fig. 3 depicts the spatial distribution of the SI persistence and the SI frequency indices for the three networks considered here. We can observe that, on average, both indices show higher values in the 2011–2017 network than in the two seasonal networks, possibly due to the higher density of the 2011–2017 network (see Table 1). Nonetheless, the overall highest values for SI persistence and frequency are found in the summer season. In terms of SI persistence, the 2011–2017 network has higher values in the coast of France and Spain, Northern Italy and the Sinai peninsula in Egypt. In winter, the nodes with higher values are located in Northeastern Italy, while non-negligible values are also found in the Balkans, Eastern coast of Egypt and the coast of France and Spain. In summer, the highest values are found in the Eastern coast of Egypt, Southern Greece and Northern coast of Spain. In terms of SI frequency, the 2011–2017 network has relatively low values of the index that are mainly located around the Aegean and Adriatic Seas, plus Tunisia and Libya. In winter, moderate values of SI frequency are found in Greece, Libya and Southern Italy. In the summer season, we observe the overall highest values concentrated in Central and Northeastern Italy, Slovenia and Croatia, with moderate values in Libya.

## Discussion

Understanding air-mass movements is a fundamental step for predicting how airborne microorganisms circulate across the planet, from their sources to their sinks via the atmosphere. Previous studies assessing microbial populations in air samples have focused on a reduced number of sampling sites and/or dates, while the choice of the sampling sites and dates is crucial in the experimental design. In this study we considered the air-mass movements over a vast geographical region (the coastline of the Mediterranean basin) for an extended period of time (7 years with a daily resolution) in order to assess spatio-temporal patterns of connectivity between different locations using the formalism of spatio-temporal networks. The proposed approach has been kept as general as possible by choosing a relatively limited set of assumptions about the parameters of the model, e.g. the duration of the backward trajectories, the hour and altitude of arrival, the spatial and temporal resolution or the yearly and monthly averaging schemes. The objective was to prove that actual air-mass connectivity patterns can be identified, and that they may be applied to the design of surveillance strategies. Nonetheless, most of these parameters can be tuned in order to fit to a particular microorganisms of interest by including knowledge on its life cycle, on which depends the emission periods, its aerodynamic diameter, on which depends the most probable airborne travelled distance or the distribution and availability of susceptible hosts, that can influence the efficacy of the disease spread. For the case study of the Mediterranean region, we identified two distinct seasonal patterns in terms of connectivity over the study period, one from May to September (here referred as summer) and the second from October to April (winter), while the yearly regime of connectivity seems to be rather constant across the years. This observation resonates with analogous conclusions drawn from studies of microbial composition in air samples, both in the Mediterranean basin^30–33^ and elsewhere^34–38^. Whether this pattern is due to a seasonal variation in the microbial sources, to the seasonal patterns of air-mass connectivity, or to a combination of both, is by itself an interesting research question. Identifying strong spreaders and receptors is a key step for designing sampling campaigns. For example, in the context of epidemic surveillance of airborne diseases, it is important to optimize the allocation of sampling sites and dates in order to increase the detection rate and to reduce the delay between arrival and first detection. In this study we identified a high-risk zone in Northeastern Italy, Croatia and Slovenia that is particularly receptive during the summer months (i.e. high SI frequency index), that correspond, at these latitudes, to the growing season of most crops. On the other hand, epidemic outbreaks starting in the coast of France and Spain, Northern Italy and the Sinai peninsula are the ones showing the highest risk of rapid diffusion across the Mediterranean basin, based on the SI persistence maps.

## Methods

### Case study region and data collection

The study region corresponds to the coast of the Mediterranean Sea, ranging approximately 1600 km from North to South and 3860 km from East to West. The temperate climate of the chosen region is strongly influenced by the presence of the Mediterranean Sea, with mild winters, hot summers and relatively scarce and seasonal precipitations. The landscape is characterized by coastal vegetation, typically shrubs and pines, and densely populated areas with intensive crop production of wheat, barley, vegetables and fruits, especially olive, grapes and citrus. In this paper, we characterize recurrent movements of air masses through the Mediterranean region by defining a grid with mesh size 74 km covering the coastline from 5 km up to 250 km inland from the coast, including the four largest islands (namely Sicily, Sardinia, Cyprus and Corsica) and the Balearic archipelago. Thus, we divided the region into *N* = 604 cells, where the centroids of the cells will be used as arrival locations of air-mass trajectories and will correspond to the nodes of the constructed network. The air-mass trajectories arriving at the prescribed locations in the period 2011–2017 with daily bases (hence $T=365 \times 7=2555$) have been computed using the Hybrid Single-Particle Lagrangian Integrated Trajectory model (HYSPLIT^45^). The HYSPLIT model has been fed with meteorological data from the Global Data Assimilation System files with a 0.5-degree spatial resolution (GDAS: https://www.ncdc.noaa.gov/data-access/model-data/model-datasets/global-data-assimilation-system-gdas) and was parametrized to return 48-h backward air-mass trajectories arriving at the prescribed locations at 12:00 GMT at an altitude of 500 m above mean sea level. A single trajectory consists of a vector containing the hourly positions (longitude, latitude and altitude) visited by the air mass before arriving at the specified location and time.

### From air mass trajectories to daily contact networks

To infer spatio-temporal networks according to air-mass trajectories, we used the formalism of graph theory, where nodes are defined as spatial points and edges are estimated using the following rule: a node *i* is connected to node *j* at time step *t* (i.e. the element ${E}_{t}(j,i)$ of the adjacency matrix ${E}_{t}$ is equal to 1) if the trajectory arriving at *i* at time *t* has crossed the polygon whose centroids is *j* over the interval $\left[t-48h, t\right]$. Since the trajectories have been computed for every *i* and every *t*, we have *T* adjacency matrices ${E}_{t}, t\in \left[0,T\right]$ of size $N \times N$, each representing a directed spatial network. (See^44^ for alternative connectivity measures).

### From daily contact networks to aggregated spatio-temporal networks

The information contained in the *T* spatial networks has been summarized by averaging all daily networks within a certain subset *S* ⊆ {0*, T* } as follows^49^:

$$\begin{gathered} E_{S} (i,j) = \sum E_{t} (i,j)/|S|, \hfill \\ t \in S \hfill \\ \end{gathered}$$

where $\left|S\right|$ denotes the cardinality of *S*. The chosen subsets are the entire discrete-time interval [0*, T*], the 7 years from 2011 to 2017 and the subsets of all days of the month of January, all the days of the month of February, etc.

### General network metrics

The constructed networks are inherently complex by the sheer amount of spatial and temporal information that they encompass. Hence, there is no easy way of representing the results either graphically or numerically, without compromising the original complexity of the networks. In this aim, we explore the topology of the networks by looking at some generic properties through the following metrics^50^, reported in Table 2:

- Density: the ratio between the sum of all edge weights and the number of all possible edges^51^,
- Transitivity (also known as clustering): the equivalent definition of density, but applied to triplets of nodes instead of pairs of nodes^52^,
- Strength correlation: in directed weighted networks, it measures the correlation between incoming and outgoing strengths, computed as the sum of the weights of the edges pointing to or from a given node. Networks with positive (resp. negative) degree correlation foster (resp. hamper) epidemic spread^53^.

**Table 2:** Network indices (Diameter, density, transitivity, degree correlation, average shortest path) calculated from the networks covering the Mediterranean region and estimated in three temporal contexts: the entire period 2011–2017, yearly time periods from 2011 to 2017 and monthly time periods.

Time period	Diameter	Density	Transitivity	degree correlation	Average shortest path
2011–2017	37.5	0.28	0.74	0.306	2.06
2011	58.5	0.17	0.68	0.132	2.63
2012	40.0	0.18	0.67	0.086	2.47
2013	40.8	0.18	0.67	0.233	2.45
2014	89.7	0.15	0.66	0.239	3.12
2015	65.4	0.16	0.66	0.236	2.75
2016	45.1	0.16	0.66	0.242	2.58
2017	44.7	0.17	0.66	0.175	2.48
January	52.5	0.13	0.64	0.168	2.69
February	114.2	0.14	0.63	0.274	4.12
March	63.4	0.13	0.63	0.265	3.10
April	64.4	0.13	0.64	0.220	3.11
May	52.1	0.12	0.64	0.180	3.08
June	72.9	0.12	0.62	0.175	3.61
July	80.3	0.10	0.60	0.061	3.47
August	61.7	0.10	0.60	0.124	3.43
September	57.7	0.10	0.61	0.167	3.45
October	102.6	0.10	0.62	0.204	4.08
November	99.3	0.11	0.63	0.378	3.93
December	59.0	0.13	0.62	0.369	2.57

Under the current framework, the weight computed between two nodes is proportional to the number of air-mass trajectories that connect them, hence higher values of the edge weight are associated to a higher connectivity between nodes. This is nonetheless incompatible with existing algorithms used to identify the shortest path between nodes since they usually consider the weight of an edge as a kind of distance or cost, hence the higher the weight, the less likely the connection between the nodes (e.g., the Dijkstra’s algorithm for weighted directed networks). Nonetheless, it suffices to transform the weights into effective distances *ED*(*i, j*) = 1 − log(*E*_*S*_(*i, j*)) in order to obtain a representation of edge weights that is coherent with the distance or cost interpretation of the search algorithms^54, 55^, after having row standardized the adjacency matrices in order to ensure that *E*_*S*_(*i, j*) ∈ [0*,* 1]*,* ∀*i, j*. After this transformation, we then compute two more indices:

- Diameter: the longest of all possible shortest paths between any two pair of nodes computed using Dijkstra algorithm^56^ (this metric does not account for the geographical distance between nodes, but only the effective distance between them),
- Shortest path length: the average of the shortest paths between any possible pair of different nodes, computed using Dijkstra algorithm^56^.

### Cut distance

The Cut distance^46^ is a particularly suitable and elegant way for comparing weighted and directed networks having the same number of nodes^57^ and it is based on the mathematical formalism of the cut distance or rectangle distance presented in^58^. We do not present here the mathematical formulation of the distance and we invite the interested reader to refer to^46^ for the details. Since the algorithm for computing the Cut distance between two networks is based on the maximisation of a certain function over all possible pairs of disjoint and complementary subsets of nodes of a network, the problem becomes quickly unfeasible as the number of nodes increases (NP-hard problem^46^). In order to solve this problem, we used a genetic algorithm from the R library GA^59^.

### Indices of network nodes relevance

Single node relevance can be computed in a multitude of ways and many indices have been proposed in the literature^60^. Here we consider seven among the most widely used indices for weighted and directed networks:

- betweenness centrality, which quantifies the number of times a node acts as a bridge along the shortest path between two other nodes^61^.
- closeness centrality, which measure the average distance of a node to all other nodes of the network^61^,
- coreness, which measures the ranking of a node after a k-shell decomposition of the network^62^,
- eigenvector centrality, which measure the tendency of highly-connected nodes to be connected to nodes with also are highly connected^63^,
- in-strength and out-strength, i.e. the sum of the weights of the edges incoming (resp. outgoing) from a node,
- strength, the sum of in-strength and out-strength.

### Susceptible–infected epidemic model

In order to model the spread of an epidemic over the constructed spatiotemporal networks, we simulated a classical SI (susceptible–infected) compartmental model^64–67^. In this model, each individual can be assigned to two distinct states: susceptible or infected. Simulations start with all nodes being susceptible, except for a single inoculated initial node. At each time step, susceptible nodes become infected if the maximum weight of their infected contacts is over a certain threshold (here arbitrary set to 0*.*1). All simulations run for 6 time steps. We simulated *N* = 604 SI epidemics for each of the three considered periods (2011–2017, summer and winter) by changing the initial infected node across all possible nodes of the networks, the rest of the parameters being constant. For every node $i \in \left\{1, \dots , N\right\}$ and every period $G \in \left\{2011-2017, winter,summer\right\}$, we recorded two values: (1) the SI persistence *P*_*G*_(*i*) that represents the maximum percentage of infected nodes at the end of the simulated epidemic that started from node *i* and spanned the period *G* and (2) the SI frequency *F*_*G*_(*i*) that represents the percentage of times that node *i* has been infected across all the epidemics ran in the *G* period.

### Software

Air-mass trajectories have been computed using the HYSPLIT^45^ software installed on local cluster https://informatique-mia.inrae.fr/biosp-cluster/cluster. All the rest of computations and graphics have been performed using the statistical software R, in particular using the packages sf^68^, ggplot2^69^, igraph^50^, GA^59^ and corrplot^70^.
